# Assessment of shelf-life and metabolic viability of a multi-strain synbiotic using standard and innovative enumeration technologies

**DOI:** 10.3389/fmicb.2022.989563

**Published:** 2022-11-04

**Authors:** Annalisa Visciglia, Serena Allesina, Angela Amoruso, Annachiara De Prisco, Raja Dhir, Peter A. Bron, Marco Pane

**Affiliations:** ^1^Probiotical Research S.r.l., Enrico Mattei, Novara, Italy; ^2^Seed Health, Abbot Kinney Blvd, Los Angeles, CA, United States

**Keywords:** plate counts, VBNC, flow-cytometry, Arrhenius model, probiotic, functional stability

## Abstract

The number of live bacterial cells is the most used parameter to assess the quality of finished probiotic products. Plate counting (PC) is the standard method in industry to enumerate cells. Application of PC implies critical aspects related to the selection of optimal nutrient media and growth conditions and underestimation of viable but not cultivable (VBNC) cells. Flow-cytometry (FC) is a culture-independent methodology having the potential to selectively enumerate live, damaged, and dead cells representing a powerful tool for in-depth monitoring of probiotic products. We monitored the shelf life of a clinical batch of a synbiotic composition PDS-08 targeting the pediatric population by means of PC and FC according to International Conference on Harmonization (ICH) pharma guidelines testing the Arrhenius model as predictive tool; PC enumeration revealed higher destruction rate than FC suggesting a faster reduction in cultivability than membrane integrity and thus a possible shift of the bacteria into a VBNC status. PDS-08 maintained acidification capability over time, when re-suspended in nutrient medium, even in samples tested sub-optimally for CFU detection (below 1 billion cells/dose). Due to similar kinetics described by the study of metabolic activity and membrane integrity, FC might be suggested as a valid tool for the study of functional stability of a probiotic product.

## Introduction

Probiotics were defined in 2001 by the World Health Organization (WHO) as “live microorganisms which, when administered in adequate amounts, confer a health benefit on the host” (Gibson et al., 2017). Consequently, international bodies such as the Food and Agriculture Organization (FAO) and WHO recommend that the product labels should include information on “minimum viable numbers of each probiotic strain at the end of the shelf-life” (FAO/WHO Guidelines, 2002). Enumeration of live probiotic bacteria may thus be considered the main evaluation tool for product feasibility, formulation, stability and quality control. Accordingly, the number of cells in finished products represents the dominant parameter used in commercial agreements, quality, and regulatory assessments. Therefore, the accurate determination of the viability of probiotics is of fundamental importance to meet the FAO/WHO guidelines (Jackson et al., 2019). In the probiotic industry, measuring viability is usually limited to enumeration of the bacteria through culture-dependent plate counting (PC) technique on nutrient agar medium (Davis, 2014). The number of colonies detected on the appropriated plates after incubation are then used to express the microbial counts as colony forming units (CFUs). Unfortunately, different probiotic strains have different optimal growth media and conditions, making the accurate enumeration of both single-and multi-strain probiotic products a challenging task. Notably, cultivability encompasses only a subset of cells considered to have the “viable” status. Indeed, evaluating cell “viability” by the PC technique likely underestimates microbial potency when cells enter the viable but not cultivable state (VBNC) during shelf life and the total number of cells present in the sample. The application of flow cytometry (FC) might overcome PC limitations, providing data on different structural and functional properties of cells which describe the viability status of the bacterial population. This can enable quantification of viability beyond cells reproductive capacity on nutrient media, providing deeper insight into the functional strain-related responses to various applications (Wilkinson, 2018; Foglia et al., 2020). The International Standard ISO 19344 – IDF 232 “Milk and milk products - Starter cultures, probiotics and fermented products - Quantification of lactic acid bacteria by flow cytometry” (ISO 19344) currently represents a benchmark for FC staining protocols based on membrane integrity, intracellular enzymatic activity, and membrane potential for the determination of bacterial cell viability. To date, the industrial use of FC for probiotics is still limited, but new evidence in methods validation (Pane et al., 2018; Foglia et al., 2020; Michelutti et al., 2020) and comparison with other possible methodologies (Gorsuch et al., 2019) is growing. The current study aimed at generating a thorough comparison of PC and FC methodologies using a Good Manufacturing Practice (GMP) clinical batch of the synbiotic formulation PDS-08 targeting the pediatric population. PDS-08 contains 9 probiotic strains and an oligofructan-enriched inulin. We monitored the shelf life of the clinical batch of PDS-08 during one-year of storage at different temperatures. According to our previously described approach (Foglia et al., 2020), the Arrhenius model for predictive microbiology was applied to data generated by FC and PC to predict the mortality of probiotic cells during storage. Alongside the evaluation of cell density through PC and FC, we introduced the study of probiotic functional stability evaluating the metabolic capacity of the lyophilized cells to acidify when inoculated in growth media. Data from enumeration by PC and FC were then compared with acidification profiles, showing that acidification trends were more comparable to FC survival kinetics than PC ones. The coupled use of PC and FC combined with metabolic activity measurements suggest that FC data provide a more accurate representation of functional stability of a probiotic product during its production and on the shelf, and consequently might be considered as valid alternative for enumeration and quality assessment method in the probiotic industry.

## Materials and methods

### Probiotic product formula

Probiotic product formula PDS-08 contain 6.3 g inulin Orafti^®^Synergy1 (Beneo, Mannheim, Germany) and the following nine probiotic strains: *Bifidobacterium animalis* subsp. *lactis* SD-Bi07-US, *Bifidobacterium animalis* subsp. *lactis* SD-CECT8145-SP, *Bifidobacterium breve* SD-BR03-IT, *Bifidobacterium breve* SD-BR632-IT, *Bifidobacterium longum* SD-CECT7347-SP, *Lactobacillus acidophilus* SD-NCFM-US, *Lacticaseibacillus casei* SD-CECT9104-SP, *Lacticaseibacillus rhamnosus* SD-GG-BE, *Ligilactobacillus salivarius* SD-LS1-IT. Labeled probiotic potency was >20 × 10^9^ viable cells per dose (6.5 g). Finished product PDS-08 was analyzed (Biolab srl, Novara, Italy) at the moment of batch release *via* flow cytometry (ISO 19344, 2015: IDF 232: 2015) which resulted in a cell count of >15 × 10^9^ Total Fluorescent Unit (TFU)/g and > 11 × 10^9^ Active Fluorescent Unit (AFU)/g; and plate count method (Internal Method 014–06) which resulted in a target cell count of >6.5 × 10^9^ CFU/g. To exclude product sample heterogeneity three random samples, withdrawn in triplicate during product manufacturing, were analyzed for total fluorescent units (TFU), and Relative Standard Deviation was <10%. Water activity of the product (a_w_) was monitored during the study to exclude any possible detrimental effects on probiotic cells or spoilage of the product due to the increase of a_w_.

### Storage conditions and stability testing according to ICH guidelines

Samples were stored and checked according to the pharma International Conference on Harmonization (ICH) Guideline Q1A (Table 1) over a period of 12 months. At each checkpoint, the number of cells (TFU, AFU and CFU), as well as the pH over 24 h and a_w_ were determined.

**Table 1:** Storage conditions according to International Conference on Harmonization (ICH) pharma guidelines Q1A for the development of new drug products.

Study	Storage condition	Minimum time needed for data submission	Control points (months)
Refrigerated condition	5°C ± 3°C	12 months	0, 3, 6, 12
Long Term	Zone II 25°C ± 2°C 60% ± 5% RH Zone IVb 30°C ± 2°C/ 75% ± 5% RH	12 months	0, 3, 6, 12
Accelerated	40°C ± 2°C 75% ± 5% RH	12 months	0, 1, 2, 3, 6

RH: Relative Humidity.

### Plate counting

Samples (dose of 6.5 g) were serially diluted in peptone saline water solution. Each dilution was plated on De Man, Rogosa and Sharpe (MRS) agar (BD Difco^™^ San Josè, CA) by inclusion technique and plates were incubated at 37°C for 72 h under microaerophilic conditions. Sample preparation and enumeration were performed following Weitzel et al. (2021) guidance for probiotic bacteria enumeration.

### Flow cytometry

FC analyses were performed using the BD Cell Viability Kit with liquid counting beads (distributed by BD Bioscience, San Josè, CA). The latter offers an easy-to-use dye combination to distinguish between live and dead cells based on assessment of cell membrane integrity. Thiazole Orange (TO) solution allows the staining of all cells while Propidium Iodide (PI) targeted damaged and dead cells. BD Liquid Counting Beads was always used as reference for the cell enumeration. Cell staining and analysis were preformed according to the ISO 19344:IDF 232 protocol with custom integration described by the method reported by Foglia et al. (2020). In order to guarantee result accuracy during the 12 months study, the flow cytometry gating procedure was kept constant at each time point. To have a reference test for enumeration, samples were preserved at-20°C and were enumerated during the study at each time point.

### Calculation and expression of results of cell enumeration

Plate count results were expressed as CFU/g (detailed in the Supplementary Material). Plates with less than 30 and more than 300 colonies were not considered in the count and only clearly visible colonies were enumerated (i.e., colonies grown on the border of the plate were not counted). Flow Cytometry results were expressed as Active Fluorescent Unit (AFU/g), non-Active Fluorescent Unit (n-AFU/g) and Total Fluorescent Unit (TFU/g). n-AFU represents the damaged and dead cells stained with PI, the non-permeant dye which only enters cells with a non-intact membrane and binds to DNA. The Total Fluorescent Unit (TFU) represents the total number of cells obtained by the sum of AFU and n-AFU cells. Damaged and dead cells (n-AFU) can be calculated also as TFU – AFU. For the stability testing, only TFU and AFU parameters have been considered. Principles for determinations of AFU and n-AFU in FC protocols using BD Liquid Counting Beads are reported in Supplementary Material.

### Evaluation of acidification activity

At each analysis time point, samples were reconstituted in 100 ml of MRS broth and incubated in a thermostatic bath at 37°C for 8 h. The acid fermentation metabolites generated serve as an indicator of metabolic or functional stability of the bacteria. Measurements of pH were recorded at the starting point (T0) and at different time points during incubation (after 2, 4, 6, 8 and 24 h) using a SevenCompact pH meter S220 (Mettler Toledo, Columbus, Ohio).

### Prediction of microbial stability using the linear Arrhenius model

A standard two-step method was used to obtain the Arrhenius model and to assess the influence of temperature on the stability PDS-08. Predictive microbiology describes the exponential loss of bacterial viability (as TFU, AFU and CFU) over time by the following equation (first order low):


(1) Nt=N0e−kt

Nt: number of viable microorganisms at t = t(x);

N0: number of viable microorganisms at t = 0;

k: destruction rate;

t: time;

By plotting ln(Nt/N0) over time (t), the destruction rate k can be determined for each stability temperature. The effect of temperature on k is described by the Arrhenius equation:


(2) k=Ae−Ea/RT

k: destruction rate (time-1);

A: frequency factor (time-1);

Ea: activation energy (J.mol-1);

R: gas constant (8,314 J.mol-1, K-1);

T: temperature (Kelvin).

By plotting ln(k) versus 1/T, a straight line is obtained, which leads to the parameters of equation (2). By extrapolation, k is obtained for any temperature included between the extremes of the stability plan. Equation (1) can be used to predict the number of viable microorganisms at any time and for any storage condition. Finally, k is related to the decimal reduction time in months (D1), the time needed for the bacterial population to be reduced by 90% that can be calculated as follows:


(3) D1=ln10/k

## Results

### Stability testing by plate count enumeration

**Figure 1:**
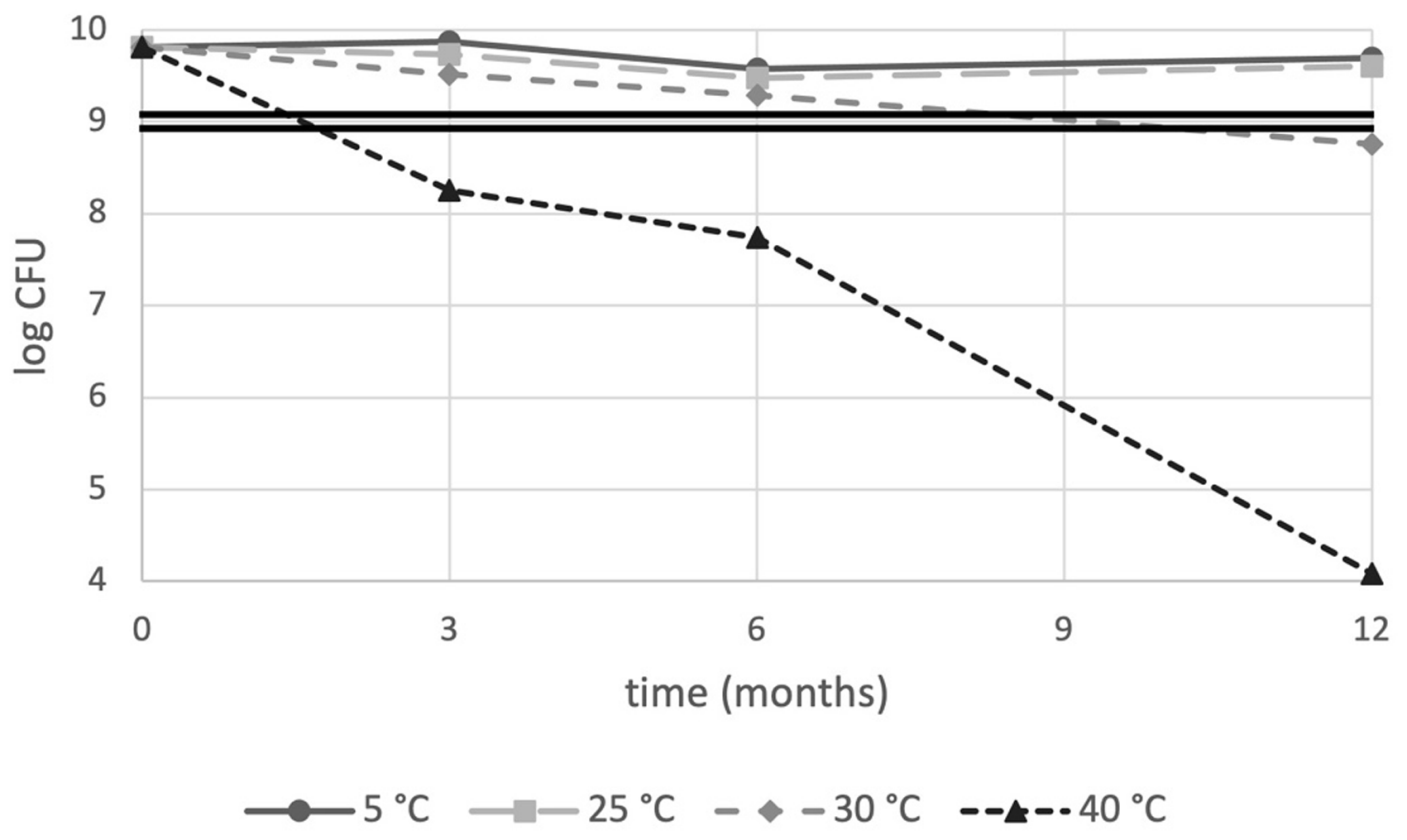
Plate count stability trends of PDS-08 over time (months) at 5, 25, 30 and 40°C. CFU data are expressed as Log numbers. The double straight line indicates the threshold value for the effective dose of 1 × 10^9^ CFU/dose, as per probiotic food guidelines.

We initially set out to perform a routine, CFU-based analysis of product shelf-life stability according to the general industry practice of PC enumeration. PC results are shown in Figure 1. Next to enumeration, Water Activity (a_w_) levels were monitored and were found below 0.25 at the extreme condition of storage at Zone IVb (30°C, 75% RH) after 12 months (data not shown). As expected, best performances were observed for lower storage temperature; sample stored at 5°C and 25°C had a survival rate of 76.9 and 61.5%, respectively, after 12 months, resulting in a total number of live cells well above 1 × 10^9^ cells which is conventionally considered the minimum effective dose for a probiotic product (Hill et al., 2014). At 30°C, cell numbers exceeded after 6 months of storage but dropped slightly below this threshold at the 12-month measurement. This drop in CFU-based viability was even more pronounced at 40°C resulting in detrimental effects after 3 months with further deterioration as time progressed (Figure 1). Taken together, the conventional industrial analysis method suggests PDS-08 can be stored for at least a year when either refrigerated or stored at room temperature, providing this does not exceed 25°C.

### Stability testing by flow cytometry enumeration

**Figure 2:**
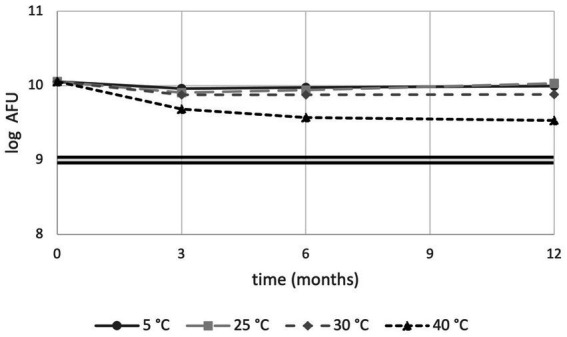
Flow cytometry stability trends of PDS-08 over time (months) at 5, 25, 30 and 40°C. AFU data are expressed as Log numbers. The double straight line indicates the threshold value of 1 × 10^9^ AFU/dose.

When stability was monitored using membrane integrity, sample stored at 5°C, 25°C, 30°C and 40°C showed 86.3, 93.8, 67.0 and 29.7% viability, respectively, after 12 months. Overall, all samples were above the cut-off limit of 1 × 10^9^ cells (Figure 2).

### CFU vs. AFU

**Figure 3:**
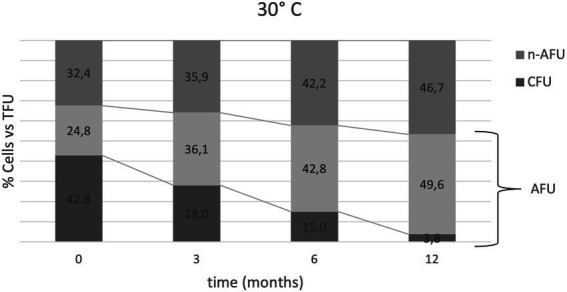
Bacterial heterogeneity distribution over time at 30°C expressed as the % of cells (CFU, AFU and n-AFU) vs. TFU (100% of the population at each time point). Relative % of AFU is the sum of AFU + CFU values; for example, t0 is represented by 42.8% CFU vs. TFU, 67.6% AFU vs. TFU and 32.4% n-AFU vs. TFU. First line from the top is representative of the AFU trend vs. time. Second line from the top is representative of CFU trends vs. time.

Predictive microbiology was applied to describe the phenomenon of lyophilized cell decay during storage at various conditions. Decrease in CFU/g (cell cultivability) and TFU/g or AFU/g (cell membrane integrity) were described by two parameters; the destruction rate (k) and the decimal reduction time (D1; section 2.6). For all tested temperatures, k values from equation (2) were substantially higher in the case of PC compared to FC enumerations (Table 2; Supplementary Figure S1), suggesting the existence of a significant subpopulation of cells that are alive but unable to form colonies. When expressed as the decimal reduction time, D1 values from equation (3) confirmed that the trend of loss in viability by CFU counts was faster than loss in membrane integrity by AFU determination. These results clearly indicate that the reduction of cultivability over time is faster than the reduction of membrane integrity at the same temperature, and that higher storage temperature are more detrimental, leading to a faster reduction of both cultivability and membrane integrity. As an example, to visualize the differences between data generated with FC and PC respectively, we plotted CFU, AFU and n-AFU over time at the temperature of 30°C in function of TFU values used as a reference (Figure 3).

**Table 2:** Bacterial/probiotic cultivability and membrane integrity destruction rate (k) and decimal reduction time (D_1_) of synbiotic samples at different temperatures.

**Temperature**	**Destruction rate k (months-1)**			**Decimal reduction time D1 (months)**		
	**CFU**	**AFU**	**TFU**	**CFU**	**AFU**	**TFU**
5°C (278.15°K)	0.0247	0.0025	0.0020	93.2	921.0	1151.3
25°C (298.15°K)	0.0468	0.0034	0.0025	49.2	677.2	932.0
30°C (303.15°K)	0.1844	0.0161	0.0141	12.5	143.0	163.3
40°C (313.15°K)	0.6983	0.1417	0.1212	3.3	16.2	19.0

CFU = Colony Forming Units; AFU = Active Fluorescent Units; TFU = Total Fluorescent Units.

### Acidification activity of lyophilized cells during storage

**Figure 4:**
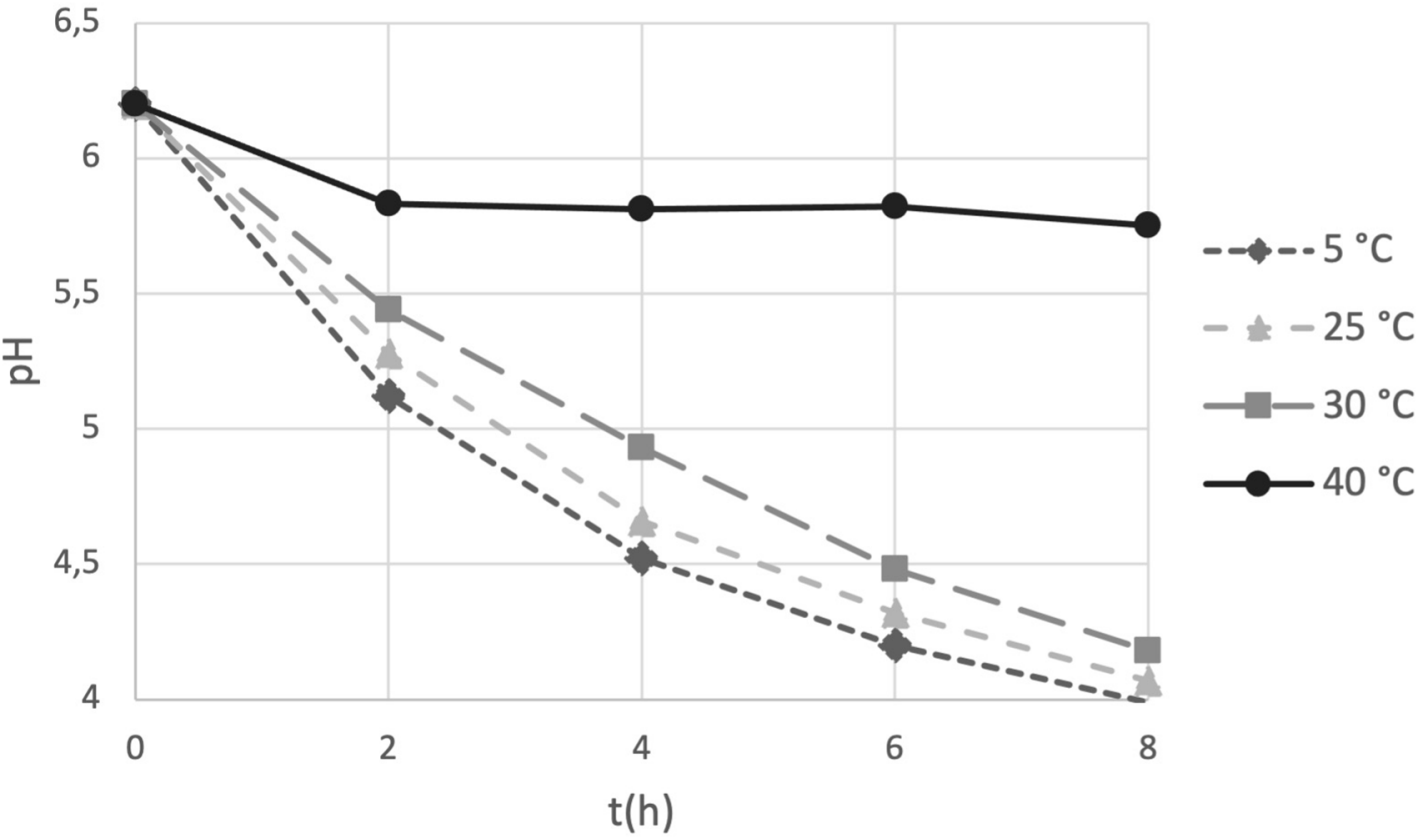
pH trends over 8 h fermentation in MRS broth by PDS-08 stored for 3 months at 5, 25, 30 and 40°C.

**Figure 5:**
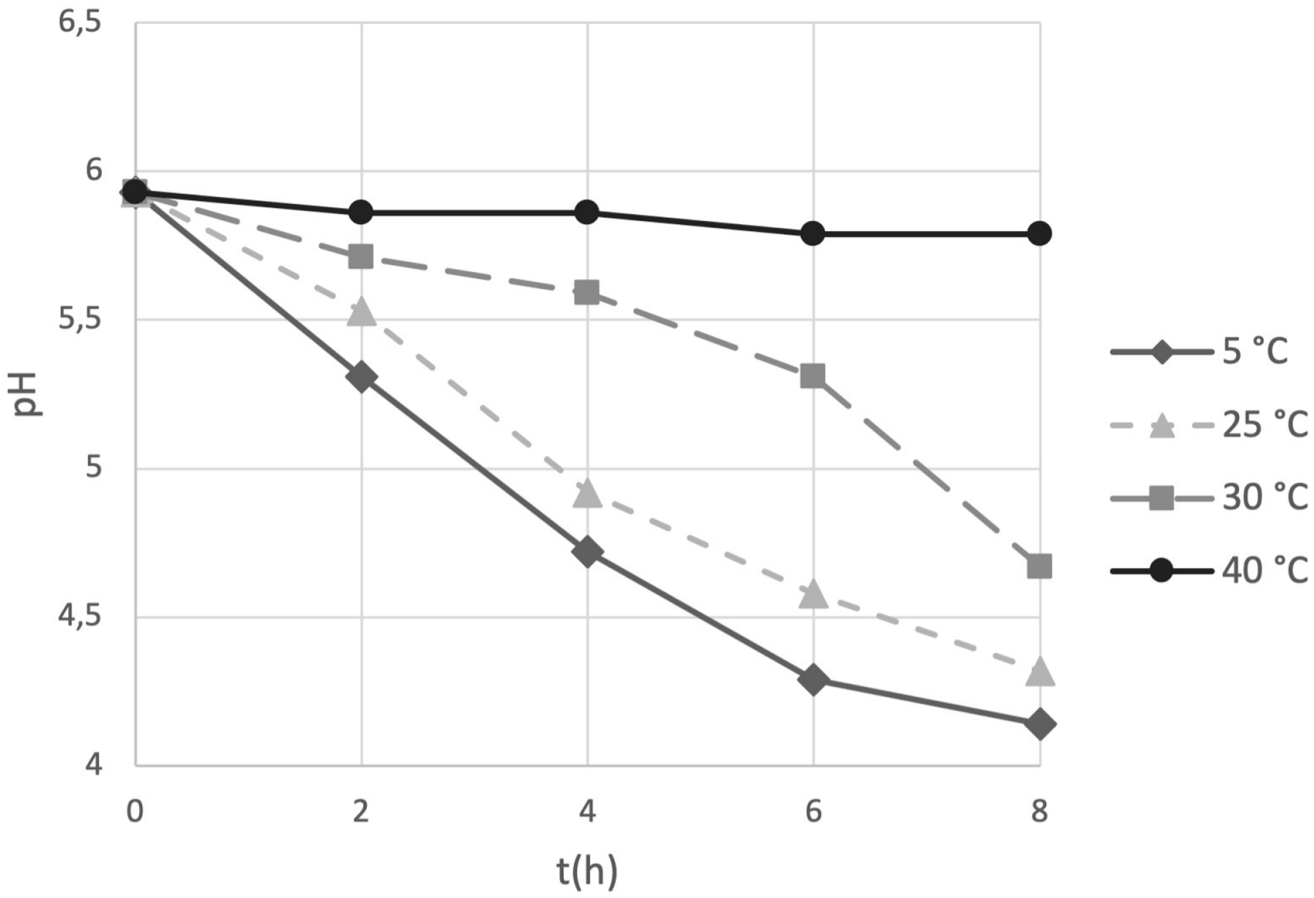
pH trends over 8 h fermentation in MRS broth by PDS-08 stored for 12 months at 5, 25, 30 and 40°C.

**Figure 6:**
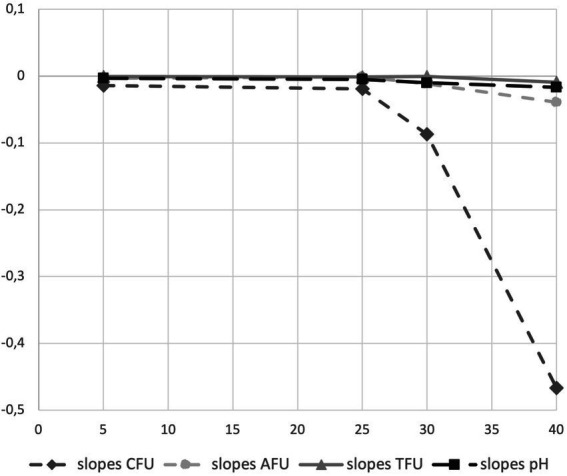
Graphical representation of acidification and enumeration (CFU, AFU and TFU) slopes obtained on clinical batch sample of synbiotic formula (PDS-08) after 12 months of storage at different temperature (5, 25, 30 and 40°C).

We introduced the study of acidification capability of cells in long-term storage as a parameter to assess the functional stability of the probiotics in the PDS-08 product. After incubating the lyophilized cells stored at different temperatures in MRS broth, pH reduction was determined at each timepoint considered for viability assessment (Table 1). To provide an efficient overview of acidification trends during time, pH kinetics obtained in the early (3rd month) and late stage (12th month) of shelf life are reported in Figures 4, 5, respectively. After 3 months of storage, samples stored at 5, 25 and 30°C retained a robust acidification capability (Figure 4). After 12 months of storage, only samples stored at 30°C recorded a marked delayed acidification while a ΔpH of 1.26 was anyway detected (Figure 5). Samples stored at 40°C showed at both the timepoints a weak acidification ability with a ΔpH of 0.2 at 3 months and 0.14 at 12 months. However, when fermentation was prolonged up to 24 h, samples stored at 40°C were able to acidify to pH 3.89 and 4.96 after 6 and 12 months of storage, respectively. To investigate the possible correlation between metabolic activity (acidification) and CFU and AFU trends, the kinetics of CFU, AFU, TFU and pH reduction were graphically represented by plotting the slopes for each parameter evaluated at 12-month control point at each temperature of storage (Figure 6). Overall, this comparison suggests that thermal stress over time affected all the parameters investigated, with higher impact on CFU followed by AFU and TFU. However, all samples retained the acidification ability during 8-24 h of fermentation in MRS even at 40°C after 12 months. From data reported in graphs represented in Figure 4, 5, regression curves have been generated to extrapolate the slopes of each pH trend (Supplementary Table S1). Plotting slopes (Supplementary Table S1) allowed to visualize the impact of temperature on the pH production by the probiotic bacteria (Supplementary Figure S2). As expected, higher temperature and longer storage time, slow down the metabolic capability of the probiotic product to acidify the medium over 8-h course (Supplementary Figure S2). Considering all the acquired information, we gathered all the enumeration and acidification slopes (Supplementary Table S2) to finally generate the graphical representation of the effect of temperature on different kinetics after 12 months of storage at different temperature (Figure 6). Finally, these group of results indicate that pH trends were more comparable to AFU trends than to the decay in CFU, suggesting the plausible contribution of a partial re-activation of VBNC cells contributing to the acidification activity used in this case as a functional marker.

## Discussion

To meet the industry guidelines (IPA 2018),^1^ probiotics products are expected to be viable at end of the declared shelf-life with the minimum documented efficacious dose (Hill et al., 2014). Many regulatory agencies rely only on culture-dependent methodologies (e.g., plate counts) to evaluate the “viability” and ensure the accuracy of label claims. However, these assays are known to underestimate microbial potency for several reasons, including the lack of a growth medium that is optimal for the target microorganisms (especially in a multi strain/species/genera consortia), and lowered cultivability when cells enter the VBNC state, and long time before data acquisition for sample incubation. The significant drop in recovered bacteria over time using plating methods could be interpreted as a loss of viable cells and is rarely interpreted as an increase in VBNC cells within the same sample. As previously reported by our group (Foglia et al., 2020), the kinetics between cultivability and a potential progression to VBNC state were monitored using a combined approach of plate counts (PC) and flow cytometry (FC) enumeration, where the first targeted the viability in terms of colonies generation and the second measured the viability in terms of cell membrane integrity depending on fluorochromes used for the analysis. Results of that study (Foglia et al., 2020) illustrate that the loss of cultivability is faster than the loss of membrane integrity within the heterogeneity of a freeze-dried single strain bacterial population. Accordingly, the hypothesis risen is that AFU cells are likely representative of the VBNC and/or dormant population plus the subgroup of cultivable cells (CFU). Consequently, VBNCs may constitute a significant portion of live cells that are consumed by the end user with potential health benefits (Blinkova et al., 2014). In this paper, we strengthen the combined use of PC and FC enumeration adding pH acidification as third pillar for monitoring functional stability (i.e., the capability of the product to acidify over time), in order to evaluate if cellular metabolism was more preferentially descriptive of FC or PC enumeration data. Since the product was constituted by fermentative micro-organisms (mainly producing acetic and lactic acids) we opted for pH as the main metabolic indicator. The synbiotic product PDS-08 was able to express primary metabolic activity up to 12 months and at different storage condition (5, 25, 30 and 40°C; Figure 4). This evidence, even in its methodological simplicity, suggests that the metabolic and functional stability is retained by the synbiotic product throughout shelf life. Interestingly, CFU decay is faster than the decreases of AFU, TFU and pH which showed similar kinetics. Besides cells viability, expression of metabolic pathways or specific effector molecules that directly interact with the host are additional mechanisms by which probiotics exert their beneficial effects (L-Chiao et al., 2013). Further research is required to verify if probiotics or candidate bacterial strains as live biotherapeutic products (LBPs) that lose cultivability (CFU) but maintain membrane integrity (AFU) can still express these molecules and to what extent. The probiotic market it is expected to exceed 85 billion USD by 2026 (Research and Markets, Probiotics – Global Market Outlook 2017–2026); the current discovery of Next Generation Probiotics (NGPs) and of LBPs reasonably will give an important boost to the market of therapeutical microorganisms in the coming years with the presence of different novel bacterial species, often strictly anerobic. In this scenario, we strongly recommend that more methodologies for cell enumeration (see Davis, 2014 for an extensive review) and function are investigated and validated. PC methodology is arduous to implement on these new species due to often-unknown growth requirements and necessity to operate in oxygen-free environment (Bircher et al., 2018). In this context, FC approach can represent a culture-independent valid tool for a real-time check of bacterial population from the early phase of a product design (e.g., strain characterization) to the latest phase (e.g., cells administration in clinical trial, finished product stability; Muller et al., 2013). Moreover, future investigation will allow the use of FC for bacterial enumeration and identification taking advantage of species-specific or even strain-specific primers (Chiron et al., 2018). As matter of fact, this work intends to open a wider research line where the first goal is the design of strain-specific and even molecule-specific markers (primers and/or antibodies). This will permit the application of our concept for the improvement of the Quality Control standards of probiotic finished products and the tailoring of probiotic formulas under the functional perspective. What we seek for the future in the Quality Control context is to be able to discriminate and quantify the single strains within a blend over time to boost to the Market of High-Quality Probiotic products; next to this, we aim at individuating probiotic-effector molecules or traits to consolidate the concept of functional stability monitoring during time both in single-and multi-strain blend. In conclusion, this paper demonstrates that a comparison of PC and FC provides a comprehensive study of the heterogeneity of a probiotic population which would be impossible by PC alone; by including a third block in the scheme that was the analysis of acidification capability used as parameter of the metabolic activity, and its similarity with FC trends, we can suggest the FC as a valid tool for the analysis of functional stability of probiotics. Accordingly, the acquisition and collection of data on bacterial samples at different stages of their cycle by means of flow cytometry is strongly encouraged. Finally, the use of the Arrhenius mathematical model was proven to be reliable also for predictive microbiology.

## Conclusion

In our previous work we presented the use of Plate Count (PC) and Flow-cytomety (FC) enumeration in a comparative approach to investigate the heterogeneity status of probiotic cells in finished products. We concluded that FC represents a valid and innovative tool for bacterial population study and enumeration and that the Arrhenius model can work as predictive model for bacterial survival during time. However, our broad scope is to elucidate the missing link between the number of probiotic cells, their status and importantly their function over a shelf-life period. In this optic, we proposed in this paper the innovative concept of functional stability, to be applied to probiotic finished product along with the monitoring of their cellular viability. The acidification capability of commercial lyophilized probiotics was selected as first and intuitive parameter to investigate. At the same time, we monitored bacterial shelf life by PC and FC followed by data elaboration with the Arrhenius model. Interestingly, we discovered that acidification kinetics by cells in storage at different temperatures were more comparable to FC survival kinetics than the ones described by PC. These results suggest that FC is a reliable tool to study the numerosity and the functional status of a probiotic population when, differently, PC limits the observation of a bacterial population purely to its cultivability. We are planning further experiments to extend this concept to the monitoring during time of probiotic effector molecules or beneficial pathways both in conventional and novel strains.

## Data availability statement

The original contributions presented in the study are included in the article/Supplementary material, further inquiries can be directed to the corresponding author.

## Author contributions

MP conceived the study. AV performed the enumeration and acidification analysis on samples in storage. SA and AA designed the set-up of the experiments and witnessed the cytofluorimetric evaluation. ADP, PB, and RD collaborated in the interpretation of the results, contributed to the writing and critically review of the paper. All authors contributed to the article and approved the submitted version.

## Conflict of interest

AV, SA, AA, ADP and MP are employees of Probiotical Research s.r.l. RD is co-founder and co-CEO of Seed Health and PB is employee of Seed Health.

## Publisher’s note

All claims expressed in this article are solely those of the authors and do not necessarily represent those of their affiliated organizations, or those of the publisher, the editors and the reviewers. Any product that may be evaluated in this article, or claim that may be made by its manufacturer, is not guaranteed or endorsed by the publisher.

## Funding

This work received no external funding.

## Footnotes

^1^ https://ipaeurope.org/wp-content/uploads/2020/03/20180523-IPA-Europe-Probiotic-Criteria-Document.pdf
