# Supplementary material for: Assessment of shelf-life and metabolic viability of a multi-strain synbiotic using standard and innovative enumeration technologies
Source: Front Microbiol. 2022 Nov 4;13:989563. doi: 10.3389/fmicb.2022.989563 (PMC9672074; doi:10.3389/fmicb.2022.989563)
Supplement: Supplementary file 1 [file Data_Sheet_1.docx]

Supplementary Material

# Supplementary Data

# Plate Count enumeration methodology

# According to ISO14461-1:2005, we expressed colony forming units (CFU) values as Weighted Mean (M)

$M=\frac{\sum Ci}{\sum Vì}$ $\frac{C1+C2+ .. +Cn}{V1+V2+ .. +Vn}$

# where:

# M: is the weighted mean CFU count per milliliter of original sample;

# Ci: are the number of colonies counted on the ith plate;

# Vi is the volume used to count the colonies on the ith plate;

# i: is the number of plates used (i = 1, 2, … n);

# n: is the total number of plates used.

# Flow Cytometry enumeration methodology

#

$$\boldsymbol{live cells}\left( \boldsymbol{AFU} \right)\boldsymbol{=}\frac{\boldsymbol{n cell in the "live" region}}{\boldsymbol{n beads region}}\boldsymbol{*}\frac{\boldsymbol{n ref beads(§)}}{\boldsymbol{test volume}}\boldsymbol{*dilution factor}$$

#

$$\boldsymbol{damaged/dead cells}\left( \boldsymbol{n-AFU} \right)\boldsymbol{=}\frac{\boldsymbol{n cell in the "damaged/dead" region}}{\boldsymbol{n beads region}}\boldsymbol{*}\frac{\boldsymbol{n ref beads(§)}}{\boldsymbol{test volume}}\boldsymbol{*dilution factor}$$

§ This value is found on the vial of BD Liquid Counting Beads and can vary from lot to lot.

# Supplementary Figures and Tables

# *Prediction of microbial stability using the linear Arrhenius model*

**Figure S1**

**Figure S2**

**Table S1**. pH slopes at different storage temperatures (5, 25, 30 and 40 °C) across four time points (0, 3, 6 and 12 months).

**Table S2.** Slopes from CFU, AFU, TFU and pH kinetics curves obtained on clinical batch sample of synbiotic formula (PDS-08) over time (12 months) at different temperatures (5, 25, 30 and 40°C).

**
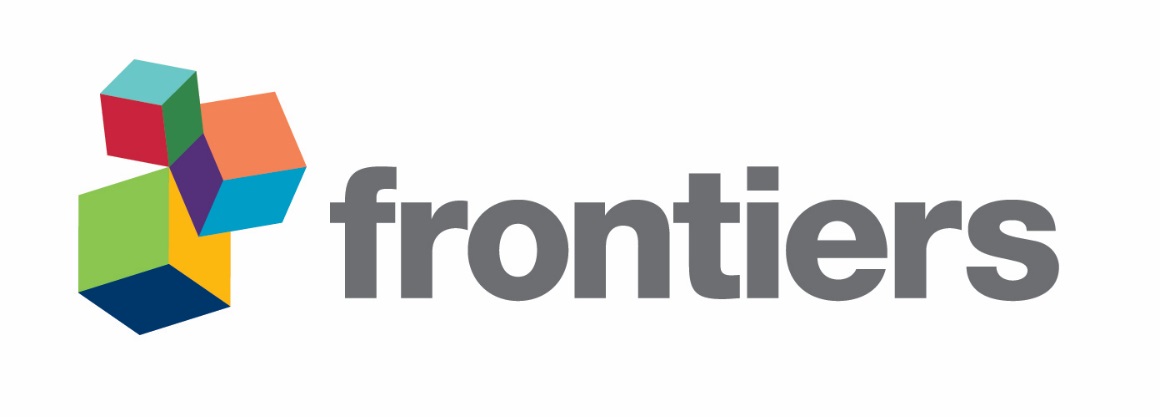
**
